# Age-associated changes in intestinal health biomarkers in dogs

**DOI:** 10.3389/fvets.2023.1213287

**Published:** 2023-08-22

**Authors:** Anna Fernández-Pinteño, Rachel Pilla, Xavier Manteca, Jan Suchodolski, Celina Torre, Anna Salas-Mani

**Affiliations:** ^1^Department of Research and Development, Affinity Petcare, L'Hospitalet de Llobregat, Spain; ^2^Gastrointestinal Laboratory, Department of Small Animal Clinical Sciences, Texas A&M University, College Station, TX, United States; ^3^School of Veterinary Science, Universitat Autònoma de Barcelona, Bellaterra, Spain

**Keywords:** fecal microbiota, canine, aging, intestinal health, nutrition

## Abstract

The gut microbiome is critical for maintaining host health. In healthy humans, the aging process is one of the main factors modulating the changes in the intestinal microbiota. However, little is known about the relationship between gut health, microbiota, and the aging process in dogs. The present study aims to explore the differences in the intestinal microbiota and intestinal health based on fecal biomarkers in a population of dogs of different ages. The study involved 106 dogs of different breeds aged between 0.2 and 15 years categorized as senior (>7 years; *n* = 40), adult (2–7 years; *n* = 50), and junior (< 2 years; *n* = 16). Fecal samples were collected during the same period at the same facilities. The analysis included the following gut health indicators: 16S rRNA gene sequencing to investigate the differences in the fecal microbiota; qPCR to determine the dysbiosis index; fecal short-chain fatty acid concentrations; fecal calprotectin; and immunoglobulin A. Beta diversity analysis revealed a significant difference with a small effect size (*p* = 0.003; *R* = 0.087) among age categories based on the unweighted UniFrac metric, but no significance was observed based on the weighted UniFrac metric or Bray–Curtis distances. There were no significant differences in the alpha diversity measures or the fecal dysbiosis index among age categories. Senior dogs had significantly higher relative abundance proportions in phyla Bacteroidota and Pseudomonadota and the genus *Faecalibacterium*, but not on qPCR analysis. At the family level, *Ruminococcaceae*, Uncl. *Clostridiales*.1*, Veillonellaceae, Prevotellaceae, Succinivibrionaceae*, and *Bacteroidaceae* abundances were higher in the senior category than in the adult and/or junior categories. Relative proportions, but not concentrations of fecal acetate, were higher in the senior category, while butyrate, isovaleric acid, and valeric acid were lower. The valeric acid concentration was significantly lower in the senior category than in the adult category. Calprotectin and immunoglobulin A levels did not differ significantly across groups. In conclusion, this study observed multiple minor changes in the fecal microbiota composition and the relative amount of short-chain fatty acids in dogs among different age groups, but studies in larger populations representative of all ages are warranted to refine the present results.

## 1. Introduction

The gut microbiome is defined as the entire collection of microorganisms living in the gastrointestinal tract, dominated by bacteria and complemented by commensal populations of fungi, viruses, archaea, and protists (1). It has the largest microbiota population in the body when compared to other colonized organs. The intestinal microbiota is estimated to be composed of 10^13^-10^14^ cells (2), and its genome is 150 times larger than the human genome.

The gut microbiome plays an important role in the physiological and pathological state of its host. It participates in multiple metabolic functions, protects against pathogens, and plays a crucial role in the immune response. These processes are critical to maintaining the health status of the host (3). The composition of the microbiota is crucial to maintaining balanced gut functionality. An imbalanced or disrupted microbiota is related to several intestinal and extraintestinal diseases (4).

There is an increasing number of studies evaluating the different elements modulating the intestinal microbiota (5–8). The main factors can be classified as external and internal, including, for example, diet and pharmacological treatments and age and genetics, respectively. In addition, pathological disorders (e.g., inflammation and type 2 diabetes) may induce deviations in the gut microbiota, causing an imbalance in the gut microbiota, defined as dysbiosis. Among all these factors, age is one of the most important variables that must be considered when studying the evolution and changes of the gut microbiota.

Aging and its relationship with gut health and gut microbiota are currently being explored in mammals, and they are already well-established in humans. Badal et al. (9) summarized the current knowledge of the human gut microbiota, considering composition, function, and metabolic products from the microbiota in relation to aging and lifespan. Changes in the human microbiota related to age have been described and are apparently associated with the host's health scenario. For example, bifidobacteria belonging to members of the genus *Bifidobacterium*, which are considered a beneficial bacterial group, decrease during the shift from middle age to old age. Contrarily, *Clostridium perfringens*, lactobacilli, enterococci, and *Enterobacteriaceae* increase during the aging process (10). In contrast to the human microbiota, bifidobacteria may not play an important role in dog gut health, according to the results reported by Masuoka et al. (10), in which bifidobacteria were only found in half of the youngest dogs and none of the adult dogs sampled, although differences in methodologies need to be considered when interpreting these results.

The existence of a core microbiota in healthy dogs has already been described (11). Moreover, Ziese and Suchodolski (12) associated shifts in the canine fecal microbiota with certain pathologies. Garrigues et al. (13) recently reviewed the development of the gut microbiota during the early stages of canine life, supporting observed changes in bacterial communities from day 2 of age to up to 52 weeks. On day 2 after birth, gut microbiota richness increases, and from day 2 to 21, Bacillota predominance is substituted by a codominance of Bacillota, Bacteroidota, and Fusobacteriota (14). Puppies in the first few weeks of life have immature microbiota, characterized by an increased dysbiosis index (DI), *Clostridium difficile* abundance, and decreased *Clostridium hiranonis* compared to adult dogs (15). Approximately after 4–6 months of age, the microbiota resembles that observed in adult dogs and remains largely stable in adulthood (13). However, the relationship between the intestinal microbiota, gut health conditions, and the aging process in older dogs is not well established. Studies in this field are needed first to understand this relationship and then to explore effective strategies to improve the quality of the aging process in dogs from this perspective.

The present study aimed to investigate the differences in the intestinal microbiota and intestinal health based on fecal biomarkers in a population of dogs of different ages, with a special focus on the senior category.

## 2. Materials and methods

All samples collected from dogs were in accordance with the 2010/63/UE directive, and any additional procedures to the dogs' daily routine were undertaken. Before the study, the protocol was shared with the Affinity Ethical Committee to ensure good practices and the animals' welfare. All dogs were housed at the Affinity Nutrition Center (Barcelona, Spain).

### 2.1. Animals

The study involved 106 multi-breed dogs (*n* = 53 male dogs; *n* = 53 female dogs) aged between 0.2 and 15.0 years (mean age = 6.3 years). The dog breeds that participated in the study were as follows: Beagle, Bichon Maltese, Jack Russell Terrier, Boxer, German Shorthaired Pointer, Poodle, Chihuahua, Cocker Spaniel, Dalmatian, German Shepherd, Spanish Water Dog, Miniature Pinscher, Andalusian Podenco, Pointer, Pomeranian, Miniature, Schnauzer, Shih Tzu, Brittany Spaniel, and Yorkshire Terrier [detailed information is in the Supplementary Table 1]. Animals were grouped into junior (J), adult (A), and senior (S) categories, considering the respective age ranges: up to 2 years, from 2 to 7 years, and over 7 years (Table 1).

**Table 1:** Descriptive information regarding the age distribution of dogs sorted by age category.

	**Junior**	**Adult**	**Senior**
	***n*** = **16**	***n*** = **50**	***n*** = **40**
Age (in years)	0.92 [0.23; 1.14]	5.00 [4.58; 7.11]	8.25 [8.15; 9.06]

Animals lived in kennels in groups of between two and seven individuals, depending on the size of the dogs. Dogs living in pairs shared an area of 15 m^2^, whereas those living in larger groups shared an area of 57–60 m^2^. All the animals had free access to the outdoor and indoor parts of their kennels, and they spent between 2 and 4 h per day in a bigger outdoor park where they could socialize with a larger group of dogs.

The water supply was *ad libitum*. Non-specific diets were manufactured and offered because of the study. Dogs were fed commercially available, dry-complete, and balanced diets adequate to their needs. Furthermore, 54% of dogs were fed a constant diet, whereas the diet was changed for 46% of dogs before and during the collection period. All diets met all requirements for protein, fat, vitamins, and minerals recommended by FEDIAF (16). Most of the dogs in the study (89%) were fed diets formulated with the following composition range: 7–8% moisture, 22–30% protein, 15–21% fat, and 2–5% fiber. The rest of the dogs (11%) were out of range because of the protein, fat, and/or fiber composition [detailed information is in the Supplementary Table 2]. Mineral and vitamin levels were similar in all the diets that were present throughout the study.

The inclusion criteria for enrollment were generally healthy animals and comprised sterilized male and female dogs of all pure breeds and ages. Based on the literature, the exclusion criteria included the presence of clinical disease or any pharmacological treatment potentially interfering with the gut microbiota and/or intestinal health. Specifically, no antibiotics, NSAIDs, PPIs, prebiotics, probiotics, or deworming treatments were administered during or 4 weeks before the sampling. None of the animals participating in this study were fed diets containing probiotics. Dogs with watery, soft, or unformed stools in three consecutive samples were excluded from the study. No clinical signs were compatible with gastrointestinal disorders (such as vomiting, regurgitation, and hyporexia) were observed in any of the animals participating in the study.

### 2.2. Fecal collection

Samples were collected during 3 months (March, April, and May) without interfering with the dogs' daily routines. Feces were collected through direct observation while the animals were kept in their kennels or outdoor recreational areas. The majority of samples were obtained during the first month, but due to the volume of samples and the physiologically atypical routines of some animals, the collection extended up to 3 months. In all cases, individualizing the animals and/or using cages to collect the samples were avoided.

One stool sample was collected per dog, and it was first scored according to an adapted five-point scale of fecal consistency (0 = watery stool; 25 = soft unformed stool; 50 = soft formed and moist stool retaining shape when being collected; 75 = hard formed stool remaining shaped but soft; and 100 = hard dry stool). The scoring system was adapted from the five-point scale previously described by Strickling et al. (17).

If fecal consistency was equal to or lower than 50, the sample was not collected, and the researchers waited for a higher fecal score. The entire stool or enough quantity to fill the sterile fecal collection tube with 250 ml was taken. External contaminants (stones, grass, sand, etc.) were discarded. All samples were processed within 4 h after the deposition. All the materials used, from sample collection to separation into aliquots, were sterile and meant for single use.

Feces were divided into two different aliquots according to the analysis to be conducted. For the microbiota analysis, between 0.5 and 1 g of feces were prepared in a 1.5-ml sterile, RNAse-free tube. For short-chain fatty acid (SCFA) analysis, calprotectin (cCP) and immunoglobulin A (IgA) were filled in one sterile stool tube with 5 g of feces. Both tubes were stored at −80°C before being sent and analyzed at the Small Animal Clinical Sciences Department at Texas A&M University (College Station, TX).

### 2.3. Fecal biomarkers

#### 2.3.1. Microbiota analysis

Illumina sequencing of the bacterial 16S rRNA genes was performed using primers 515F (5′-GTGYCAGCMGCCGCGGTAA) (18) to 806RB (5′-GGACTACNVGGGTWTCTAAT) (19) at the MR DNA laboratory (Shallowater, TX, USA).

Sequences were processed and analyzed using the Quantitative Insights Into Microbial Ecology 2 (QIIME 2) (20) v 2021.8 pipeline. The raw sequences were uploaded to the NCBI Sequence Read Archive under the BioProject identification PRJNA901473. In brief, the sequences were demultiplexed, and the ASV table was created using DADA2 (21). Before downstream analysis, sequences assigned as chloroplast, mitochondria, and low abundance ASVs, containing < 0.01% of the total reads in the dataset, were removed. All samples were rarefied to even sequencing depth, based on the lowest read depth of samples, to 21,025 sequences per sample.

Alpha diversity was measured with the Chao1 (richness) and Shannon diversity metrics within QIIME2. Beta diversity was evaluated with the unweighted and weighted phylogeny-based UniFrac (22) distance metric (measures that consider phylogenetic information) and the Bray-Curtis distance metric and visualized using Principal Coordinate Analysis (PCoA) plots, generated within QIIME2.

The dysbiosis index (logDNA) is based on a validated algorithm that considers a panel of eight bacterial groups identified by a Quantitative PCR assay for total bacteria, *Faecalibacterium* spp., *Turicibacter* spp., *Escherichia coli, Streptococcus* spp., *Blautia* spp., *Fusobacterium* spp., and *Clostridium hiranonis* (23). A DI of < 0 was defined as normal, a DI between 0 and 2 was defined as mild to moderate microbiota shift, and a DI of >2 was considered significant dysbiosis.

#### 2.3.2. Intestinal health indicators

Concentrations of SCFAs (acetate, propionate, butyrate, isobutyric acid, isovaleric acid, and valeric acid) in feces were measured using a stable isotope dilution gas chromatography-mass spectrometry (GC-MS) assay with some modifications (24). To consider the difference in water content between fecal samples, the final concentrations of fecal SCFAs were adjusted by fecal dry matter (DM) and expressed as μmol/g of fecal DM (25).

The fecal IgA (mg/g) was quantified by using the commercial ELISA kit for canine IgA determination (Bethyl Laboratories, Montgomery, TX, USA). The quantification of the fecal cCP (ng/g) was also analyzed by using the ELISA kit previously validated in dogs (26, 27).

### 2.4. Statistical analysis

For the statistical analysis of microbiota data, an analysis of similarity (ANOSIM) test within the PRIMER 7 software package (PRIMER-E Ltd., Luton, UK) was performed to analyze significant differences in microbial communities and the size effect (*R*-values between 0 and 1; a higher R-value indicates a larger size effect) among age groups (J, A, and S). All datasets were tested for normality by performing the Shapiro–Wilk test (JMP Pro 11, SAS Software Inc.). The Kruskal–Wallis test was performed (Prism v. 9, GraphPad Software Inc.), followed by a *post-hoc* Dunn's multiple comparison tests, to determine the age group differences in bacterial taxa (including phylum, class, order, family, genus, and species). All *p*-values were adjusted for multiple comparisons using Benjamini and Hochberg's False Discovery Rate (28) at each taxonomic level, and an adjusted *p*-value of < 0.05 (*q*-value) was considered statistically significant.

For the general statistical analysis, the model included demographic information as an explanatory variable and fecal indicators as response variables. The primary explanatory variable was age, which was grouped into the three different categories described. The potential explanatory variables included the following: body weight, sex, “feeding routine,” “housing,” and “breed.” Two categories were established for feeding routines: rotation (R), if animals were changing the type of diet in periods shorter than 2 weeks, and stable (S), if animals were eating the same diet for at least 4 weeks. No animals had a change of diet in the 2–4 week range. Each animal was only assigned to one category during the whole study. Housing was the variable defined to identify the three different buildings where animals were allocated. Finally, as for the breed, the dogs were categorized into two groups: beagles and non-beagle dogs. The different breeds were not statistically analyzed due to the high number of different breeds but due to the low number of dogs within each breed; however, the dog size was introduced in the statistical analysis to reduce the heterogeneity of dogs participating in the study. The response variables considered in the general statistical analysis were as follows: fecal consistency; SCFA concentration (acetate, propionate, butyrate, isobutyric acid, isovaleric acid, valeric acid, and total SCFA); SCFA relative amounts (acetate, propionate, butyrate, isobutyric acid, and isovaleric acid); calprotectin; immunoglobulin A; alpha diversity (Chao 1 and Shannon diversity); and finally, from qPCR, total bacteria, *Faecalibacterium, Turicibacter, Streptococcus, E. coli, Blautia, Fusobacterium, Clostridium hiranonis, Bifidobacterium, Bacteroides, Lactobacillus*, and dysbiosis index.

For the general statistical analysis, a first summary statistic was performed, in which quantitative variables were analyzed using the mean and standard deviation. Qualitative variables were tested using relative and absolute frequencies. The existence of differences among age groups was tested by performing the appropriate tests (ANOVA and Kruskal–Wallis test), considering the equality among groups' null hypothesis. The compliance of the application criteria was assessed by performing the Shapiro–Wilk normality test. The relationship between quantitative variables was analyzed using Spearman's correlation.

To analyze the relationship between the age categories and fecal markers, the appropriate linear model was considered, including potential explanatory variables such as body weight, sex, feeding routines, and housing breed. Estimated means (emmeans) for age categories were calculated using the adjusted model. Pairwise *post-hoc* comparisons were also performed, and the model validation was analyzed by performing a graphical residual analysis. For the general statistical analyses, differences were considered significant with a *p*-value of < 0.05.

## 3. Results

### 3.1. Quantitative real-time PCR analysis and dysbiosis index

**Figure 1:**
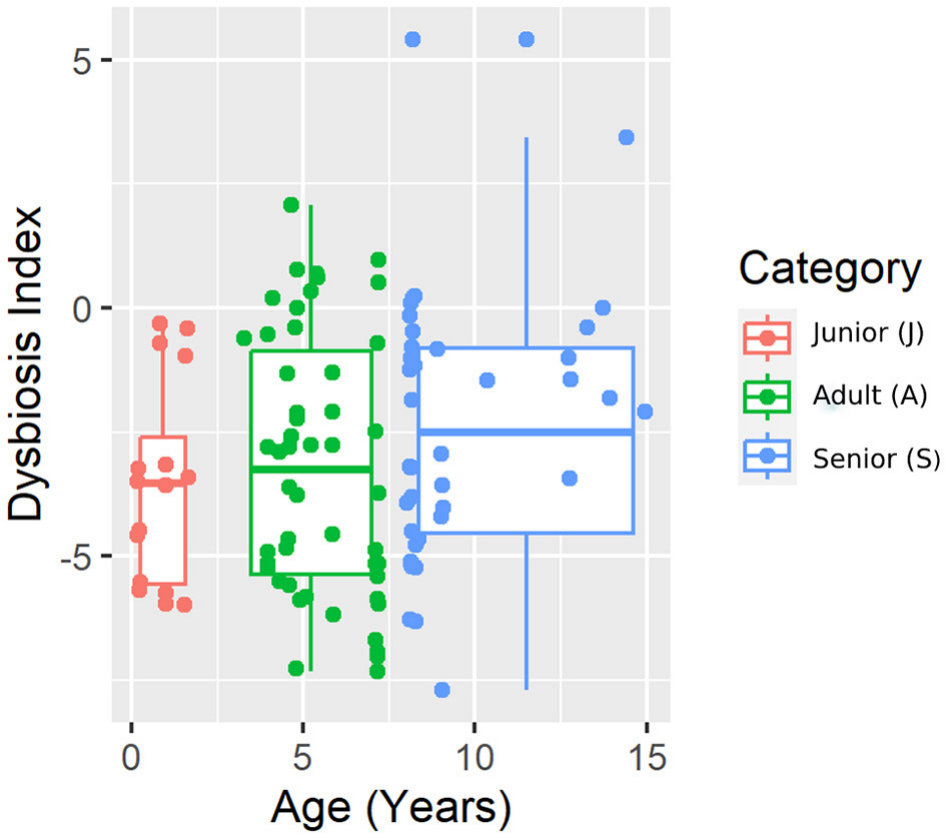
A representative plot of the dysbiosis index (DI) split by age category.

The results from the qPCR analysis are summarized in Table 2. *Bifidobacterium* abundance was different between J and A, being higher in A. However, no difference was found when considering S vs. J and A. The calculated dysbiosis index was not different among the age categories (Figure 1). In J, 0 of 16 (0%) dogs had a DI of >0, while in A and S, 8 of 50 (16%) and 5 of 40 (13%) dogs had a DI of >0.

**Table 2:** Results of the quantitative real-time PCR (abundance expressed as LogDNA) and values of the calculated dysbiosis index (ratio).

	**Junior**	**Adult**	**Senior**	
	**emmeans** ±**SE**	**emmeans** ±**SE**	**emmeans** ±**SE**	***p*** **-value**
Dysbiosis index	−4.51 ± 0.642	−3.98 ± 0.370	−3.49 ± 0.518	0.468
Universal LogDNA	10.9 ± 0.067	11.0 ± 0.038	11.0 ± 0.054	0.723
*Faecalibacterium*	5.43 ± 0.285	5.44 ± 0.164	5.76 ± 0.230	0.476
*Turicibacter*	7.23 ± 0.237	7.74 ± 0.137	7.53 ± 0.192	0.125
*Streptococcus*	4.57 ± 0.406	5.10 ± 0.235	5.31 ± 0.328	0.362
*E.coli*	4.89 ± 0.441	5.12 ± 0.254	5.26 ± 0.356	0.814
*Blautia*	10.7 ± 0.088	10.5 ± 0.051	10.6 ± 0.071	0.124
*Fusobacterium*	8.91 ± 0.189	8.81 ± 0.109	8.91 ± 0.152	0.774
*Clostridium hiranonis*	6.80 ± 0.91	6.86 ± 0.523	6.81 ± 0.073	0.729
*Bifidobacterium*	4.09 ± 0.530^b^	6.04 ± 0.306^a^	5.35 ± 0.428^a, b^	0.004
*Bacteroides*	5.72 ± 0.195	5.84 ± 0.113	6.00 ± 0.158	0.525
*Lactobacillus*	4.88 ± 0.454	5.58 ± 0.262	6.11 ± 0.367	0.118

Different superscript letters indicate significant differences between groups (p-value < 0.05).

### 3.2. 16s rRNA sequencing

**Figure 2:**
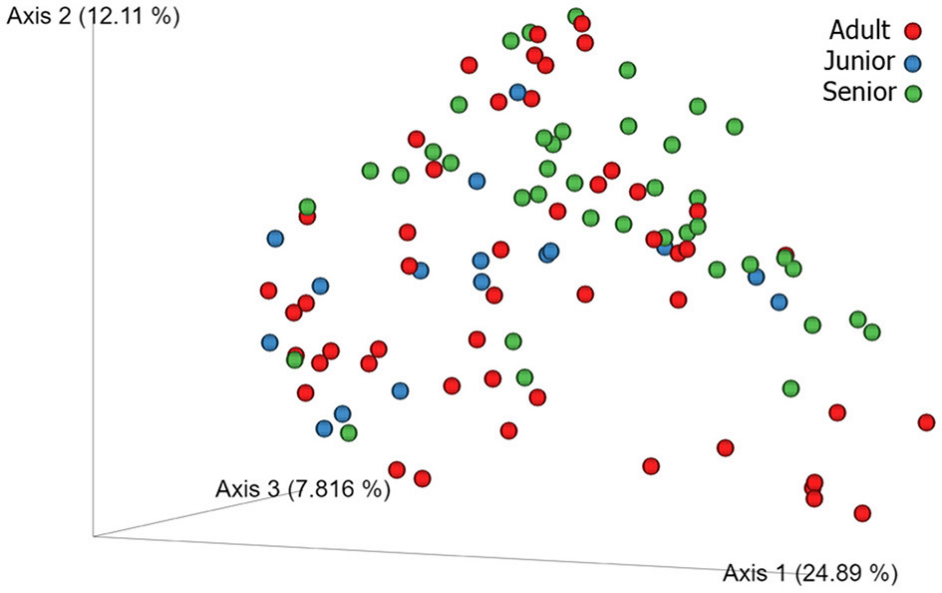
3D beta diversity patterns of canine fecal microbiota comparing Adult (red), Junior (blue) and Senior (green) categories based on unweighted UniFrac distance.

**Figure 3:**
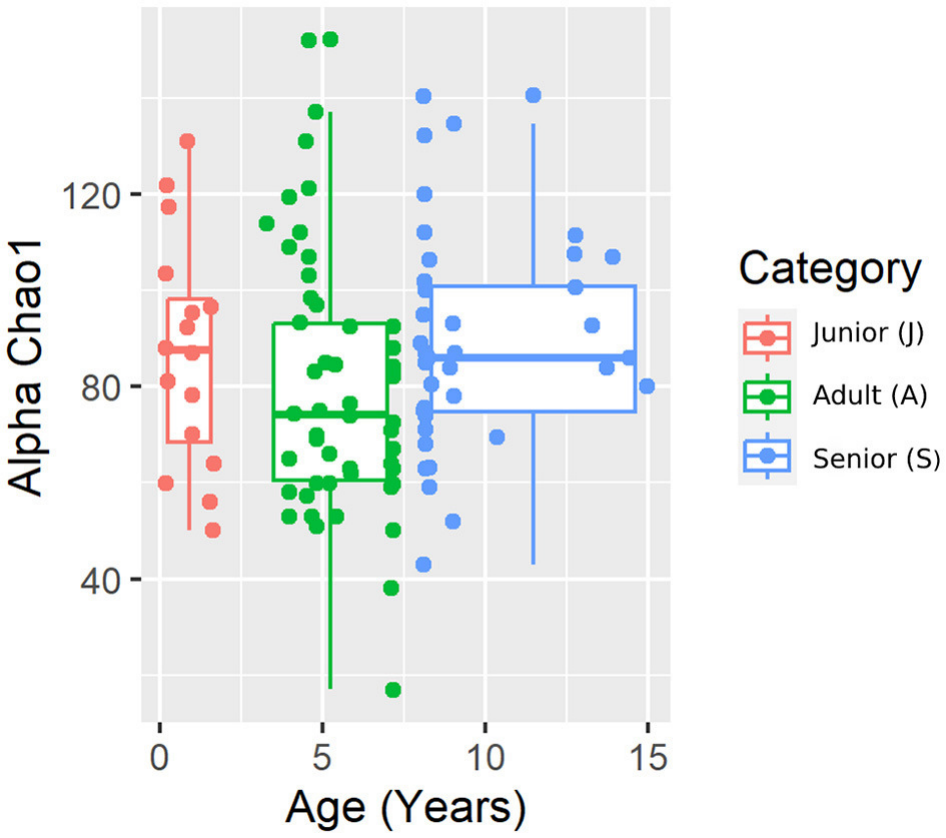
Chao 1 alpha diversity index plot by category.

**Figure 4:**
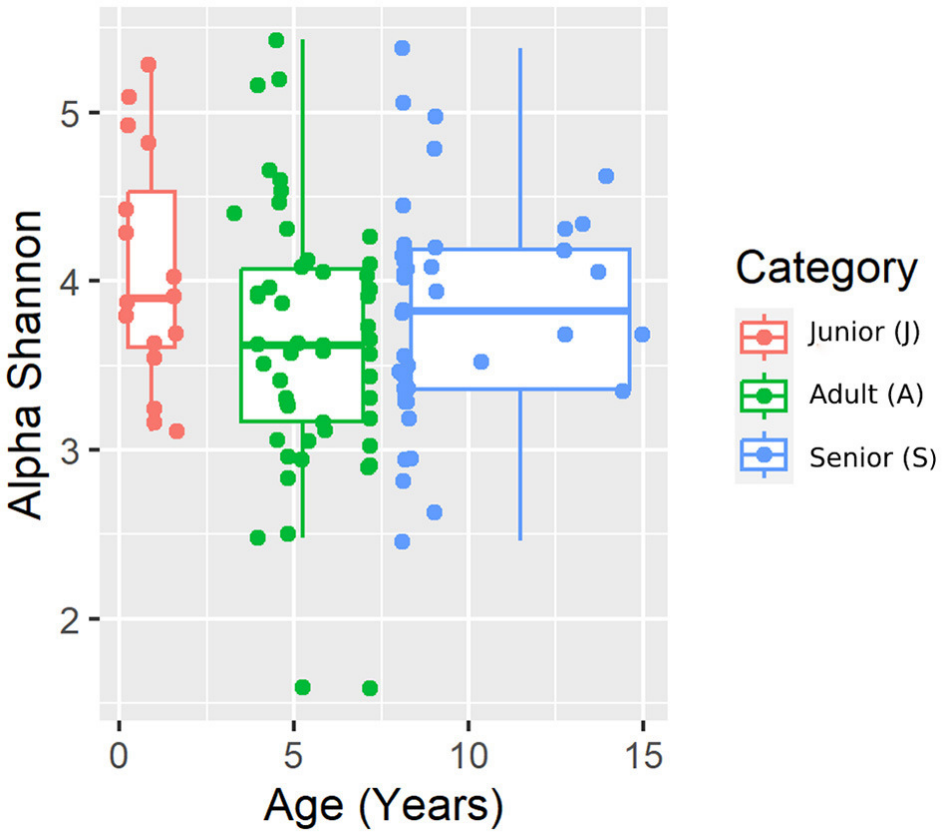
Shannon alpha diversity index plot by age category.

A total of 3,856,065 quality bacterial 16S rRNA sequences were obtained from the 106 fecal samples analyzed. The range count per sample was between 21,030 and 60,827 (median: 33,688 and mean: 36,378). The beta diversity was analyzed by the weighted and unweighted UniFrac distances and the Bray–Curtis distance. The unweighted UniFrac analysis of similarities, which only considers the presence or absence of individual taxa, revealed significant differences in the microbial communities among age categories (PCoA plot shown in Figure 2). However, no significance was observed based on weighted UniFrac metric or Bray–Curtis distances (Table 3). Alpha diversity indices (Chao1 and Shannon) were not significantly different among the age groups (Figures 3, 4).

**Table 3:** Results of beta diversity analysis including unweighted UniFrac, weighted UniFrac, and Bray–Curtis distances.

	**Senior vs. Adult vs. Junior**		**Senior vs. Adult**		**Senior vs. Junior**		**Junior vs. Adult**	
	***R***	***p*** **-value**	***R***	***p*** **-value**	***R***	***p*** **-value**	***R***	***p*** **-value**
Unweighted UniFrac	0.087	0.003	0.071	0.003	0.150	0.018	0.070	0.117
Weighted UniFrac	−0.023	0.759	0.047	0.024	−0.145	0.993	−0.127	0.970
Bray–Curtis	0.001	0.459	0.037	0.028	−0.077	0.863	−0.045	0.715

The table shows the sample statistic (R) and the significance level of the sample statistic (p-value < 0.05), considering the three age categories and the pairwise test comparison.

#### 3.2.1. Phylum taxonomic level

When the bacterial relative abundance was studied at the tax level, the major phylum identified was Bacillota, followed by Actinomycetota, Fusobacteriota, Bacteroidota, and Pseudomonadota. The same distribution, considering relative abundance, was described in the three age categories. Significant differences were found for the phylum Bacteroidota and Pseudomonadota, in which S had a higher abundance than A and J (Table 4). However, the corrected q-value did not reach significance.

**Table 4:** Fecal microbiota composition (% of relative abundance from rarefied data) at the phylum level split by age category.

**Phylum**	**Junior**		**Adult**		**Senior**		**Junior vs. Adult vs. Senior**	
	**Median**	**Range**	**Median**	**Range**	**Median**	**Range**	***p*** **-value**	***q*** **-value**
Bacteroidota	0.06^a, b^	0–3.67	0.53^a^	0–11.01	1.74^b^	0.21–12.02	0.036	0.087
Bacillota	89.60	77.20–97.52	85.28	71.62–95.08	82.02	64.30–88.92	0.052	0.087
Actinomycetota	6.87	1.47–14.05	5.35	1.66–14.13	8.20	2.60–15.70	0.569	0.569
Fusobacteriota	1.20	0.15–10.52	5.08	0.13–20.34	3.60	0.89–27.9	0.145	0.181
Pseudomonadota	**0.01** ^**a**^	**0–0.60**	**0.27** ^**a, b**^	**0–1.73**	**0.28** ^**b**^	**0.04–2.14**	**0.019**	**0.087**

Significantly different results are indicated in bold (p-value < 0.05). Superscript letters indicate differences between age categories.

#### 3.2.2. Family taxonomic level

**Figure 5:**
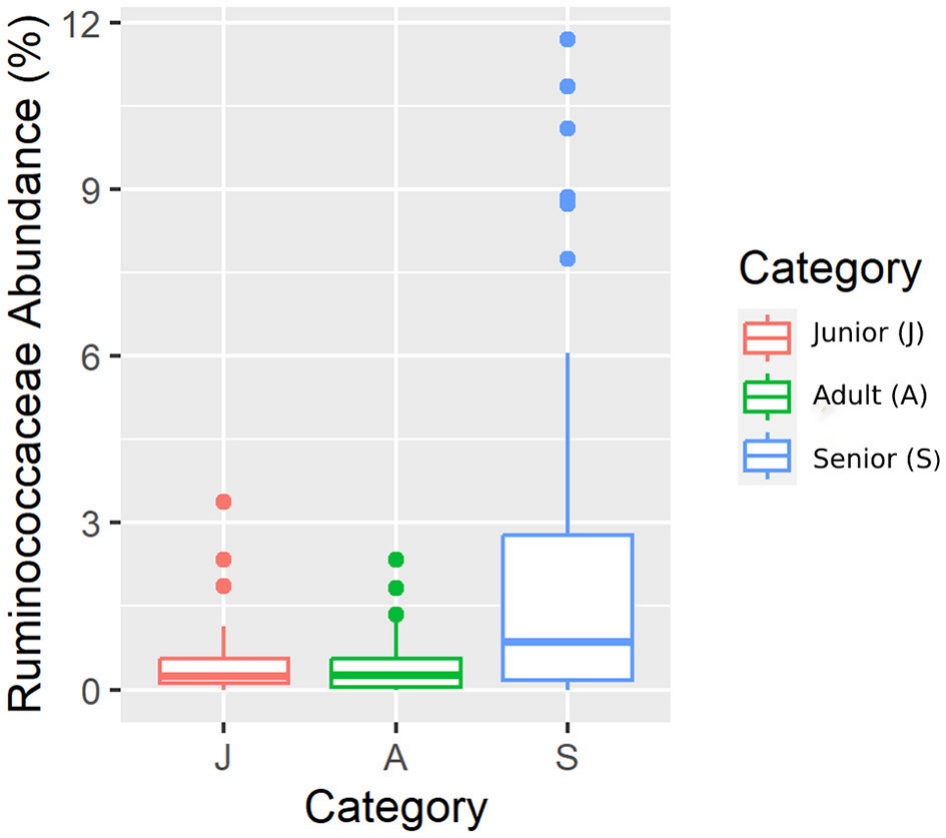
A box plot representation of the relative abundance from rarefied data of the family *Ruminococcaceae* family by age category.

**Figure 6:**
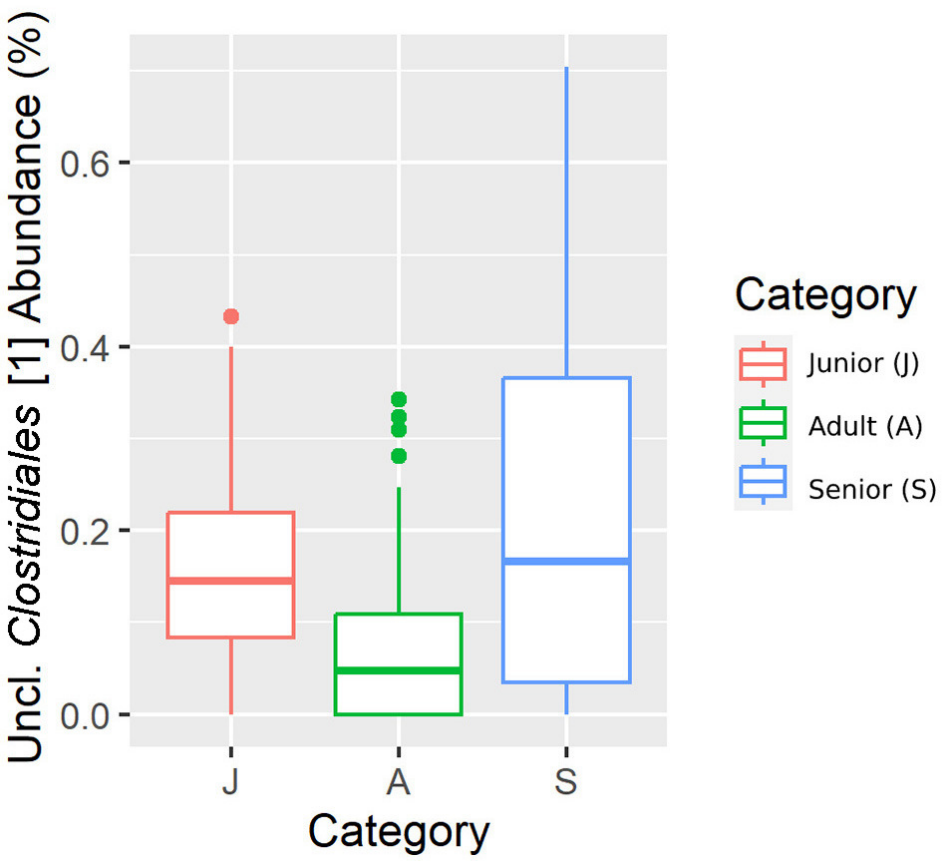
A box plot representation of the relative abundance from rarefied data of the Uncl. *Clostridiales*.1 family by age category.

**Figure 7:**
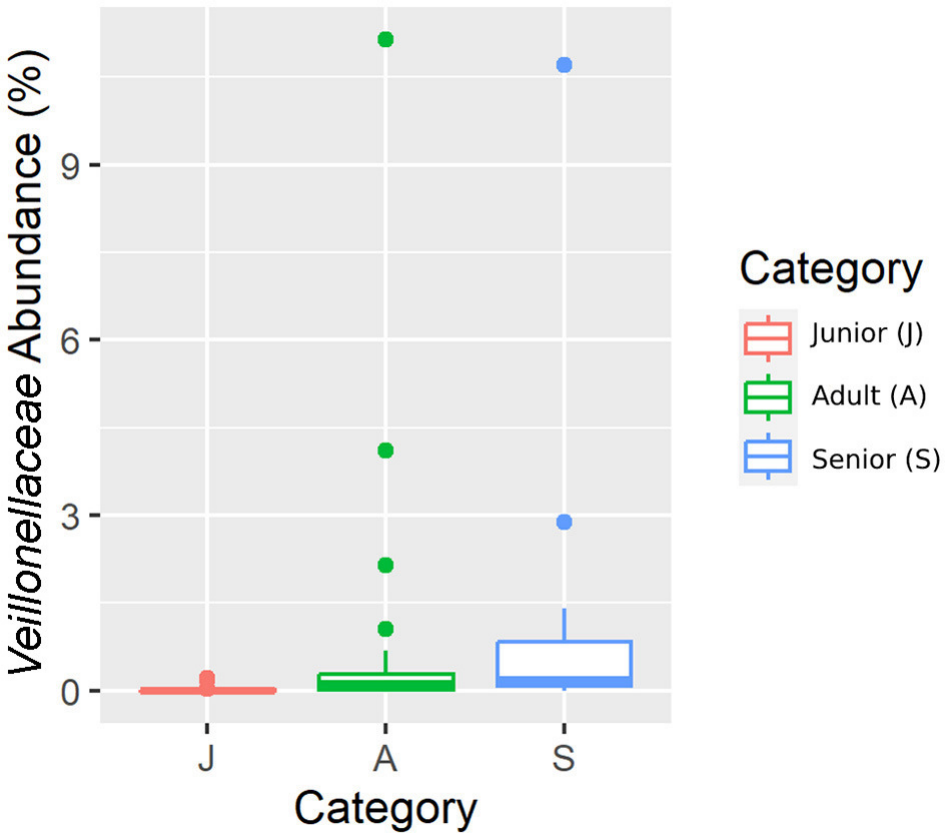
A box plot representation of the relative abundance from rarefied data of the *Veillonellaceae* family by age category.

**Figure 8:**
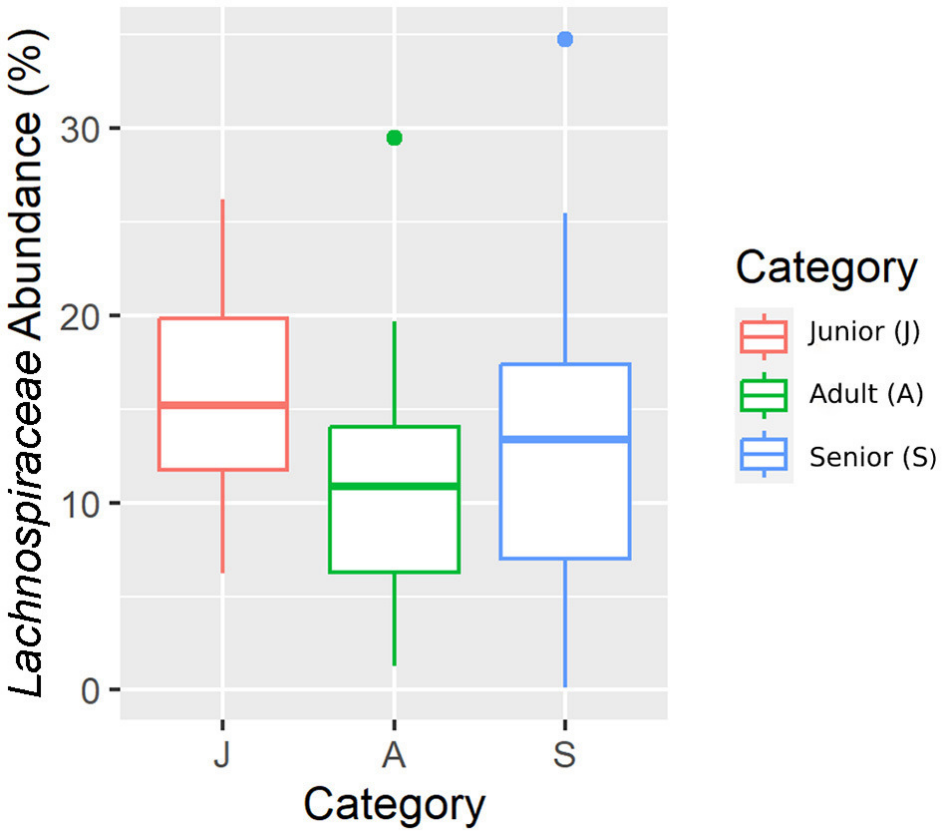
A box plot representation of the relative abundance from rarefied data of the *Lachnospiraceae* family by age category.

Falling into the main phylum (Bacillota), *Ruminococcaceae*, Uncl. *Clostridiales*.1*, Veillonellaceae*, and *Lachnospiraceae* families were significantly different among the age categories (Figures 5–8). Specifically, the abundance of *Ruminococcaceae* and Uncl. *Clostridiales*.1 was higher in the S category than in the A category, and *Veillonellaceae* showed a higher abundance in the S category than in the J category. *Lachnospiraceae* were significantly higher in the J category than in the A and S categories and did not differ significantly. Within the Bacteroidota phylum, the relative abundance of *Bacteroidaceae* and *Prevotellaceae* was different among the age categories; *Bacteroidaceae* was higher in the S category than in the A and J categories, and *Prevotellaceae* was higher in the S category than in the A. For Pseudomonadota, the relative abundance of *Succinivibrionaceae* was higher in the S category than in the A category. Finally, the *Bifidobacteriaceae* family (Actinomycetota) was significantly higher in the A category than in the J category; however, the adjusted q-value was not significant (Table 5).

**Table 5:** Fecal microbiota composition (% of relative abundance from rarefied data) at the family level split by age group.

**Family**	**Junior**		**Adult**		**Senior**		**Adult vs. Junior vs. Senior**	
	**Median**	**Range**	**Median**	**Range**	**Median**	**Range**	***p*** **-value**	***q*** **-value**
*Bifidobacteriaceae*	**0.06** ^**b**^	**0–0.70**	**1.43** ^**a**^	**0–13.44**	**0.18** ^**a, b**^	**0–4.78**	**0.024**	**0.066**
*Coriobacteriaceae*	6.73	1.17–14.05	3.84	0.68–13.01	7.10	0.88–15.69	0.204	0.264
*[Paraprevotellaceae]*	0.03	0–1.08	0.03	0–1.24	0.15	0–1.05	0.115	0.211
*S24-7*	0	0–0.5	0.1	0–10.8	0	0–7.8	0.201	0.264
*Bacteroidaceae*	**0.03** ^**a**^	**0–1.33**	**0.07** ^**a**^	**0–0.44**	**0.73** ^**b**^	**0.21–3.09**	**< 0.001**	**0.009**
*Prevotellaceae*	**0** ^**a, b**^	**0–0.74**	**0** ^**a**^	**0–0.19**	**0.03** ^**b**^	**0–6.93**	**0.016**	**0.049**
*Erysipelotrichaceae*	10.01	2.18–20.99	22.31	2.25–83.44	5.58	3.71–52.05	0.199	0.264
*Clostridiaceae*	34.47	8.34–46.61	24.94	3.07–65.36	39.12	5.27–57.80	0.628	0.641
Uncl. *Clostridiales*.1	**0.17** ^**a, b**^	**0–0.39**	**0.05** ^**a**^	**0–0.28**	**0.19** ^**b**^	**0.05–0.72**	**0.008**	**0.049**
*Peptostreptococcaceae*	1.17	0–11.38	0.70	0–8.22	0.23	0.03–1.46	0.094	0.189
*Lachnospiraceae*	**15.13** ^**b**^	**6.16–24.89**	**7.44** ^**a**^	**1.17–19.26**	**13.81** ^**a, b**^	**3.66–23.45**	**0.011**	**0.049**
*Turicibacteraceae*	7.86	0.14–25.78	1.48	0.11–13.28	2.56	0.33–8.39	0.167	0.263
*Streptococcaceae*	0.23	0–16.38	1.02	0–22.00	0.19	0–14.31	0.641	0.641
*Lactobacillaceae*	0.69	0–67.59	0.09	0–20.13	1.01	0–47.93	0.242	0.296
*Ruminococcaceae*	**0.31** ^**a, b**^	**0–3.45**	**0.25** ^**a**^	**0.01–2.19**	**1.99** ^**b**^	**0–11.46**	**0.014**	**0.049**
*Peptococcaceae*	0.32	0–0.91	0.23	0–1.82	0.17	0–0.89	0.570	0.627
Uncl. *Clostridiales*.2	0.32	0–0.51	0.29	0–0.71	0.22	0–0.48	0.494	0.572
*Veillonellaceae*	**0** ^**a**^	**0–0.18**	**0.01** ^**a, b**^	**0–0.2**	**0.15** ^**b**^	**0–1.25**	**0.009**	**0.049**
*Fusobacteriaceae*	1.20	0.15–10.52	5.08	0.13–20.34	3.60	0.89–27.90	0.145	0.246
*Succinivibrionaceae*	**0** ^**a, b**^	**0–0.51**	**0** ^**a**^	**0–0.11**	**0.03** ^**b**^	**0–0.18**	**0.013**	**0.049**
*Alcaligenaceae*	0	0–0.20	0.14	0–1.60	0.10	0–2.10	0.051	0.124
*Enterobacteriaceae*	0	0–0.04	0.02	0–1.59	0.03	0–1.01	0.094	0.189

Significantly different results are indicated in bold (p-value < 0.05). Superscript letters indicate differences between age categories.

#### 3.2.3. Genus taxonomic level

The two predominant genera identified in the dogs' population belonged to the phylum Bacillota, *Uncl. Clostridiaceae*.1, and *Allobaculum*. Within Bacillota, *Faecalibacterium*, and Uncl. *Clostridiales*.3 showed the highest values in S, being significantly different when compared with A (*p* = 0.006 and 0.008, respectively). The relative abundance observed in the genus *Faecalibacterium* was 18.6 times higher in the S category than in the A category. However, the difference in *Faecalibacterium* was not confirmed by a targeted qPCR. Within this genus, the relative abundance of *F. prausnitzii* was 18.4 times higher in the S category (median 1.3% of sequences) than in the A category (median 0.07%; *p* = 0.006 and *q* = 0.030). Detailed information at the species level is in the Supplementary Table 3. In relation to *Phascolarctobacterium* abundance, S was higher than the A and J categories (*p* = 0.011). Although the S category also showed higher values for *Ruminococcus* when compared with A (*p* = 0.035), the adjusted q-value did not reach significance. Contrarily, the abundance of *Eubacterium*, Uncl. *Erysipelotrichaceae*, and Uncl. *Lachnospiraceae* was not significantly different when the S category vs. the J and A categories were considered. However, J showed significantly higher values for the three genera when compared with the A category (*p* = 0.005, *p* = 0.001, and *p* = 0.002). Specifically, J showed 8.6 and 7.2 times more relative abundance in *Eubacterium* and Uncl. *Erysipelotrichaceae* genera, respectively, compared with A. Considering the Uncl. *Peptostreptococcaceae* genus, the relative abundance was higher in J than in S and A categories (*p* = 0.009).

The relative abundance of *Bacteroides* (phylum Bacteroidota) was 24.3 and 10.4 times higher in the S category than in the J and A categories, respectively (*p* < 0.001). *Prevotella* (*Prevotellaceae* family) was higher in S than in the A category (*p* = 0.016); however, the adjusted q-value was not significant. The relative abundance of *Fusobacterium* (phylum Fusobacteriota) was higher in the S category than in the A category when considering only the significance of the *p*-value (*p* = 0.045), but this was not confirmed by targeted qPCR. Detailed information at the genus level is in the Supplementary Table 4.

### 3.3. Fecal SCFAs, IgA, and cCP

Valeric acid was significantly lower in the S category than in the A category. However, the concentrations for total SCFA were numerically higher (but not significant) in the S category and lower in the A and J categories (Table 6). Acetate, butyrate, isovaleric acid, and valeric acid reached significant results when they were analyzed as relative percentages (Table 6). In the *post-hoc* analysis, the S category showed higher values than A for acetate (*p* < 0.001) and lower values for butyrate, isovaleric acid, and valeric acid (*p* = 0.001, *p* = 0.045, and *p* = 0.026, respectively). For isovaleric acid, the S category had lower values than J. Finally, no significant differences among age categories were found for the fecal IgA and cCP concentrations (Table 7).

**Table 6:** Fecal SCFA values expressed as concentration (μmol/g DM) and relative percentages (%) reported by age category.

**Fecal SFCA**	**Junior**	**Adult**	**Senior**	
	**emmeans** ±**SE**	**emmeans** ±**SE**	**emmeans** ±**SE**	***p*** **-value**
**Concentration (**μ**mol/g DM)**				
Acetate	169 ± 26.1	210 ± 15.0	249 ± 21.1	0.061
Propionate	10.2 ± 1.449	12.4 ± 0.837	14.0 ± 1.171	0.149
Butyrate	52.8 ± 8.25	71.4 ± 4.76	61.2 ± 6.66	0.079
Isobutyric acid	6.91 ± 1.109	7.92 ± 0.640	7.27 ± 0.896	0.634
Isovaleric acid	9.72 ± 1.404	8.16 ± 0.811	6.69 ± 1.134	0.249
Valeric acid	**4.55** **±2.06**^**a, b**^	**8.59** **±1.19**^**a**^	**4.70** **±1.66**^**b**^	**0.049**
SCFA total	254 ± 34.7	319 ± 20.0	343 ± 28.0	0.130
**Relative percentages (%)**				
Acetate	**69.4** **±1.78**^**a, b**^	**66.0** **±1.02**^**a**^	**72.9** **±1.43**^**b**^	**< 0.001**
Propionate	4.17 ± 0.342	4.18 ± 0.197	4.23 ± 0.276	0.986
Butyrate	**18.8** **±1.552**^**a, b**^	**22.0** **±0.896**^**a**^	**17.3** **±1.253**^**b**^	**0.003**
Isobutyric acid	2.63 ± 0.304	2.62 ± 0.175	2.22 ± 0.246	0.358
Isovaleric acid	**3.43** **±0.359**^**a**^	**2.69** **±0.207**^**a, b**^	**2.01** **±0.290**^**b**^	**0.011**
Valeric acid	**1.56** **±0.507**^**a, b**^	**2.44** **±0.293**^**a**^	**1.38** **±0.410**^**b**^	**0.043**

Significantly different results are indicated in bold (p-value < 0.05). Superscript letters indicate differences between age categories.

**Table 7:** Fecal cCP and IgA values by age category.

	**Junior**	**Adult**	**Senior**	
	**emmeans** ±**SE**	**emmeans** ±**SE**	**emmeans** ±**SE**	***p*** **-value**
cCP (ng/g)	4.67 ± 0.564	3.90 ± 0.326	3.79 ± 0.456	0.420
IgA (mg/g)	11.5 ± 3.14	11.1 ± 1.81	12.2 ± 2.54	0.935

Different superscript letters indicate significant differences between groups (p-value < 0.05).

### 3.4. Correlations among qPCR, IgA, cCP, and SCFA

Considering significant correlations (rho) equal to or >0.3, *Faecalibacterium* abundance correlated positively with propionate and valeric acid concentrations, while *Fusobacterium* correlated positively with isobutyric concentration. *Clostridium hiranonis* correlated positively with isovaleric acid percentage, and *Bifidobacterium* correlated positively with valeric acid concentration. This genus correlated positively with the acetate relative percentage, while the correlation with the butyrate relative percentage was negative. *Bacteroides* genus correlated positively with valeric acid concentration and its relative percentage. IgA concentrations correlated negatively with isobutyric, isovaleric, and valeric acid concentrations. This indicator also correlated negatively with isobutyric and valeric absolute percentages. Correlation indexes are detailed in the Supplementary Table 5.

## 4. Discussion

Our study supports previously published results on the relationship between the aging process and gut health and gut microbiota in dogs. Notably, we included a wider age range (between 0.2 and 15 years old) than previous studies on puppies and adult dogs. In addition, we studied our dog population from different perspectives based on the analysis of fecal microbiota together with other intestinal health biomarkers to broadly understand the overall changes. Our main focus for the discussion of the results is the senior life stage, the period when the quality of life is threatened by the aging process itself. Exploring gut health and microbiota based on fecal indicators may help find specific diet interventions to improve the quality of life of aging dogs.

To compare the selected indicators along the different life stages, this study included dogs with specific diet needs because of their age, which may be considered a confounder to understanding the results among the age categories. However, the diet, together with body weight, sex, feeding routine, housing, and breed, were considered potential explanatory variables for the general statistical analysis.

### 4.1. Microbiota results

Bacillota, Actinomycetota, Fusobacteriota, Bacteroidota, and Pseudomonadota were the five main phyla found in the fecal samples analyzed in our studied population of dogs using 16S rRNA gene sequencing. Previous studies described that most bacterial sequences identified in the gut belong to these five different phyla (29–31). In this regard, previous literature may have used the old phylum terminology for Bacillota, Actinomycetota, Fusobacteriota, Bacteroidota, and Pseudomonadota, which were previously called Firmicutes, Actinobacteria, Fusobacteria, Bacterioidetes, and Proteobacteria, respectively (32). Although the names have recently changed, these phyla have been previously identified as the main contributors to gut microbial composition in dogs. More specifically, Bacillota, Bacteroidota, and Fusobacteriota were described as the three predominant phyla in the healthy canine fecal microbiome in previous studies (33, 34). However, in our study, within the three main bacterial groups, the phylum Actinomycetota was found instead of Bacteroidota. Interestingly, the distribution of relative abundance was the same across the three categories. The low abundance in Bacteroidota phyla could be because of obesity and gastrointestinal disease (35–37). Although Actinomycetota is present in the small intestine of healthy animals, the relative abundance found in feces is lower, and its increase may be associated with pathological conditions (29). Considering the health status of the population studied, obesity and gastrointestinal pathologies do not explain the low numbers obtained, and the differences may be explained by the analysis itself. The influence of methodological aspects in 16S rRNA gene sequencing on the results is important to consider, so different studies are difficult to compare. Previous studies have reported significant differences in the abundance of bacterial groups based on variations in labs and methodologies (38–40).

The *Bifidobacteriaceae* family is an important bacterial group for humans. Considering the results for *Bifidobacterium* in the qPCR and the sequencing analysis, this genus was present in all three age categories (senior dogs, adult dogs, and puppies), being more representative in adults and less representative in puppies. Although intestinal microbiota composition has been studied in different animal species, including dogs, the transition of the intestinal microbiota with age has not been thoroughly investigated. Masuoka et al. (10) studied a different age population, from pre-weanling puppies to senior dogs, to evaluate age-dependent differences and changes in the intestinal microbiota by employing a culture-based method. Bifidobacteria were detected in puppies but were not present when analyzing dogs older than 3 years old (adult dogs and senior dogs). Based on the literature on human beings, the presence of this genus in the three categories should be understood as positive. Bifidobacteria is the most prevalent bacteria in infants and adults, and it is presumed to promote health benefits in the host (41). However, the extrapolation of the results among animal species should be interpreted carefully. *Bifidobacterium* abundance in the qPCR correlated positively with valeric acid concentration and acetate relative percentage. Positive correlations between Bifidobacterium and acetate have been previously described (42, 43), although further studies investigating links with valeric acid may be warranted.

Senior dogs had a higher relative abundance of the families *Ruminococcaceae, Veillonellaceae, Bacteroidaceae*, and *Lachnospiraceae* (phylum Bacillota). Previous studies have found decreases at this level in dogs with inflammatory bowel disease (IBD) (25, 44), but there is no clear relationship with aging. Senior dogs also had higher values in the relative percentage of acetate, one of the main products of bacterial fermentation. These two findings are in the same direction as previously published data; there is a well-known positive correlation between the bacterial groups classified in Bacillota phyla and the production of SCFA (25). However, our results supported the fact that relative percentages of butyrate and valeric acid were significantly lower in the population of senior dogs. The relative percentages are logically influenced by the high numbers in the acetate concentration. Focusing on the valeric result, the relative percentage was supported by the concentration result, which was also lower in the senior group. These findings may be interpreted as negative considering gut health since SCFA production provides an appropriate pH environment to maintain healthy microbiota, inhibits the growth of pH-sensitive pathogenic bacteria, and preserves gut integrity (45). Moreover, valeric acid has been positively associated with the gut-brain axis and cognitive function in mice (46). Therefore, senior dogs may not benefit from the mechanisms surrounding the production of SCFA that promote intestinal health and positively contribute to cognitive function.

Although *Faecalibacterium* genera were not significantly different in the qPCR analysis, *Faecalibacterium prausnitzii* was found to be higher in older dogs, which may be considered a minor positive change for this aged population. This discrepancy may be caused by the fact that *F. prausnitzii* falls into the *Faecalibacterium* genera together with other species. Another possible reason could be that *Facecalibacterium* is frequently undetected in dogs during sequencing due to its low abundance, but it could be detected using qPCR. In addition, *Faecalibacterium* qPCR was significantly positively correlated with propionate and valeric acid concentrations, which is positive for gut health. The absence of this bacterium has been associated with gastrointestinal disorders in dogs (36) and humans (47). Focusing on the species level, *F. prausnitzii* has been clearly associated with increased fiber utilization and different fiber types in the diets of dogs (8, 48). However, the diet's fiber content was not considered a cause of the higher abundance described in the S category for *F. prausintzii*. In our study, only 5% of the dogs were out of the defined fiber composition range (2–5%), and they were similarly distributed in the three different age categories (J = 2/106; A = 2/106; and S = 1/106). From this information, considering the importance of *F. prausnitzii* within the *Faecalibacterium* genera, we could state that the difference found in senior dogs is a positive change in the microbiota population related to aging.

At the genus level, the two predominant groups identified belong to the phylum Bacillota (*Uncl. Clostridiaceae*.1 and *Allobaculum*). These findings support previously published data on dogs, in which major taxa fell within the Bacillota group and the bacterial class Clostridia was considered the most abundant taxon (29). In our study, the *Fusobacterium* genus (within the Fusobacteriota phylum) was present in all the age populations and was specifically higher in dogs over 7 years old. This finding can be considered positive for the microbiome of senior dogs since this genus has previously been related to a healthy intestinal microbiome in dogs (13).

### 4.2. Beta diversity, alpha diversity, and dysbiosis index

For beta diversity, unweighted UniFrac was significantly different among age categories when ANOSIM analysis was performed. However, the size effect as estimated by the *R*-value was extremely small, and other distance metrics were not significant. The changes found in our population, and especially the categories defined, may not be enough to change the weighted UniFrac and Bray–Curtis beta diversity. Another possible explanation for the observed differences in the unweighted UniFrac but not in the weighted UniFrac and Bray–Curtis beta diversity could be attributed to the fact that these two metrics consider relative abundances instead of only the presence/absence of different taxa. Furthermore, alpha diversity analysis measured by the Chao1 and Shannon indexes was not significantly different among the age groups. Our findings are consistent with previous studies. You and Kim (31) studied different individual traits in healthy dogs and also reported no differences at the age level when studying microbial diversity patterns. Interestingly, none of the dogs belonging to the junior category had a moderate or significant change in the index (DI > 0). Blake et al. (15) showed that the DI increased in younger puppies, but many dogs at 9 weeks and most dogs after 6 months of age had normal DI. In our study, the junior age ranged from 2 to 19 months; therefore, our data are consistent with previous studies, as most dogs over 6 months are expected to have a DI of < 0. From those results, we could state that, in our study, small changes in the microbiota may have occurred due to aging. To conclude with the microbiota data, the performance of the Kruskal–Wallis test for assessing differential abundance between groups could be a limitation. This statistical test considers the overall abundance between groups but not the frequency of organism identification in samples within a group. Further statistical tests for assessing differential abundance between groups (e.g., ANCOM and DESeq) could complement the information analyzed by the Kruskal–Wallis test.

### 4.3. Fecal SCFAs, IgA, and cCP

Fecal cCP and IgA concentrations in feces have been studied as non-invasive biomarkers of gut health in adult dogs. Fecal cCP is an indicator of intestinal inflammation in dogs (49, 50), whereas IgA is associated more with the gut immune response (51). In the present study, the fecal concentration of cCP and IgA was not related to the aging process since no differences were observed among the three age categories. Based on the literature, aged mice and humans did not show a clear relationship between age and the concentration of mucosal IgA (52). Zaine et al. (53) reported no significant differences in IgA fecal values in senior dogs compared with adults and puppies. However, 5-month-old puppies had lower IgA concentrations than adult dogs. Fecal cCP concentrations were recently correlated with age in healthy humans in Korea (54), but to our knowledge, the results on cCP concentrations in healthy dogs of different ages are not consistent. The lack of clear reference values for fecal cCP and IgA considering the different age stages is a clear limitation to thoroughly understanding our results in relation to age and the SCFA correlation found in our study. However, in our study, age did not change the intestinal inflammation status or gut immune response based on the two indicators analyzed.

### 4.4. Main features

Considering the overall results of the present study, minor changes in the intestinal health biomarkers analyzed were found among the junior, adult, and senior categories. The microbiota community and SCFA production showed age-dependent differences. However, the IgA and cCP were not significantly different across the three categories.

Our age category definition may be a limitation to thoroughly understanding the continuous changes of the microbiome in relation to the aging process. Significantly, no clear consensus exists about this classification in animal research or veterinary medicine (55). The definition of age categories may reduce the sensitivity of understanding whether different ages equally affect gut health and microbiota. Thus, a larger number of animals representative of all age categories may help define more categories to increase the sensitivity of these first results.

In addition, the extrapolation of this trial in a field setting study may help better explore effective strategies, such as dietary interventions, in the dog population.

## 5. Conclusion

In conclusion, in this study, the canine gut microbiota and certain SCFAs showed minor variations among age groups in dogs. Other intestinal health biomarkers (IgA and cCP) were similar among age groups. Considering all the parameters, we can state that, once the microbiota becomes stable in healthy dogs, minor changes occur during the aging process.

The modulation of these minor changes, considering the intestinal microbiota and short-chain fatty acid production, could improve the overall gut health during aging in dogs.

## Data availability statement

The datasets presented in this study can be found in online repositories. The names of the repository/repositories and accession number(s) can be found below: https://www.ncbi.nlm.nih.gov/, PRJNA901473.

## Ethics statement

The animal study was reviewed and approved by Affinity Petcare Ethical Committee.

## Author contributions

AF-P, AS-M, and CT designed and performed the study. AF-P and RP analyzed the results and prepared the tables and graphs for inclusion in the manuscript. AF-P wrote the manuscript. Overall, all authors contributed to the paper, discussed its results, reviewed the drafts, and approved the final version submitted for publication.
