# Supplementary material for: Age-associated changes in intestinal health biomarkers in dogs
Source: Front Vet Sci. 2023 Aug 22;10:1213287. doi: 10.3389/fvets.2023.1213287 (PMC10481537; doi:10.3389/fvets.2023.1213287)

Supplementary material

Supplementary table 1 (S1). Detailed information of the number of dogs per each breed distributed by categories (junior, adult, senior).

Breed	Age Category			Total
	Junior	Adult	Senior	
Beagle	11	16	11	38
Bichon Maltese		3	2	5
Jack Russell Terrier			3	3
Boxer			2	2
German Shorthaired Pointer			1	1
Poodle			2	2
Chihuahua	4	9	2	15
Cocker Spaniel		1	2	3
Dalmatian			2	2
German Shepherd			2	2
Spanish Water Dog			2	2
Miniature Pinscher			1	1
Andalusian Podenco			2	2
Pointer			1	1
Pomeranian		1	2	3
Miniature Schnauzer		4	2	6
Shih Tzu		2		2
Brittany Spaniel			1	1
Yorkshire Terrier	2	14		16

Supplementary table 2 (S2). Detailed product composition of the 6 pet food products out of the composition range defined in the manuscript (7-8% moisture, 22-30% protein, 15-21% fat and 2-5% fiber). The animal count for each type of product and its age category are included in the table. The analytical constituent values out of the defined composition range are marked with an asterisk (*).

**Product type**	**Product composition - Analytical constituents (%)**				**Age Category**		
	**Moisture**	**Protein**	**Fat**	**Fiber**	**Junior (n=16)**	**Adult (n=50)**	**Senior (n=40)**
Adult light	8	27	9,5*	4,8			3
Senior	7,5	27	12,2*	2,5			2
Renal veterinary diet	7,5	14,5*	17,5	3			2
Colitis veterinary diet	8	23	12*	6,5*			1
Gastroenteric veterinary diet	7,5	25,5	12,8*	1,3*	2		
Weight control veterinary diet	7,8	34*	10,5*	11,5*		2	

Supplementary table 3 (S3). Microbiome results summary for taxa relative abundance (% of relative abundance from rarefied data) including phylum, family and genus splitted by age category. The results that are significantly different are marked in bold (p-value < 0.05). Superscript letters are indicating the differences between the age categories.

			Junior		Adult		Senior		J vs A vs S	
**Phylum**	**Family**	**Genus**	**Median**	**Range**	**Median**	**Range**	**Median**	**Range**	**p-value**	**q-value**
Actinomycetota			6.87	1.47-14.05	5.35	1.66-14.13	8.20	2.60-15.70	0.569	0.569
	Bifidobacteriaceae		**0.06^{b}**	**0-0.70**	**1.43^{a}**	**0-13.44**	**$0.18^{a,b}$**	**0-4.78**	**0.024**	**0.066**
		Bifidobacterium	0.06^{b}	0-0.70	1.43^{a}	0-13.44	$0.18^{a.b}$	0-4.78	0.024	0.081
	Coriobacteriaceae		6.73	1.17-14.05	3.84	0.68-13.01	7.10	0.88-15.69	0.204	0.264
		Adlercreutzia	0.12	0-0.79	0.16	0-0.39	0.31	0-0.75	0.496	0.586
		Collinsella	5.62	0.87-13.11	2.86	0.16-12.26	6.17	0.44-14.98	0.181	0.272
		Slackia	0.59	0.17-1.62	0.07	0-0.80	0.52	0-0.76	0.078	0.169
**Bacteroidota**			**$0.06^{a,b}$**	**0-3.67**	**0.53^{a}**	**0-11.01**	**1.74^{b}**	**0.21-12.02**	**0.036**	**0.087**
	Bacteroidaceae		**0.03^{a}**	**0-1.33**	**0.07^{a}**	**0-0.44**	**0.73^{b}**	**0.21-3.09**	**<0.001**	**0.009**
		Bacteroides	**0.03^{a}**	**0-1.33**	**0.07^{a}**	**0-0.44**	**0.73^{b}**	**0.21-3.09**	**<0.001**	**0.013**
	[Paraprevotellaceae]		0.03	0-1.08	0.03	0-1.24	0.15	0-1.05	0.115	0.211
		[Prevotella]	0.03	0-1.08	0.03	0-1.24	0.15	0-0.98	0.165	0.272
	Prevotellaceae		**$0^{a,b}$**	**0-0.74**	**0^{a}**	**0-0.19**	**0.03^{b}**	**0-6.93**	**0.016**	**0.049**
		Prevotella	**$0^{a.b}$**	**0-0.74**	**0^{a}**	**0-0.19**	**0.03^{b}**	**0-6.93**	**0.016**	**0.061**
	S24-7		0	0-0.5	0.1	0-10.8	0	0-7.8	0.201	0.264
		Uncl. S24-7	0	0-0.47	0.07	0-10.84	0.02	0-7.80	0.201	0.291
Bacillota			89.60	77.20-97.52	85.28	71.62-95.08	82.02	64.30-88.92	0.052	0.087
	Clostridiaceae		34.47	8.34-46.61	24.94	3.07-65.36	39.12	5.27-57.80	0.628	0.641
		Clostridium	**0^{b}**	**0-0**	**0.02^{a}**	**0-0.74**	**$0^{a.b}$**	**0-0.29**	**0.025**	**0.081**

		Uncl. *Clostridiaceae*.1	34.30	8.1-45.29	24.41	2.62-65.36	37.47	4.95-57.28	0.603	0.672
		Uncl. *Clostridiaceae*.2	0.23	0-1.32	0.54	0-2.57	0.51	0-3.52	0.265	0.333
	Erysipelotrichaceae		10.01	2.18-20.99	22.31	2.25-83.44	5.58	3.71-52.05	0.199	0.264
		Allobaculum	5.05	0.88-15.05	20.87	0.27-83.42	3.34	1.49-51.93	0.752	0.785
		Catenibacterium	1.21	0.18-8.65	0.07	0-9.21	0.24	0-2.71	0.124	0.221
		Coprobacillus	0.07	0-0.25	0	0-1.54	0.03	0-0.93	0.237	0.315
		[Eubacterium]	**1.2[b]**	**0.21-4.58**	**0.14[a]**	**0-1.28**	**0.36[a,b]**	**0-7.05**	**0.005**	**0.037**
		**Uncl. *Erysipelotrichaceae***	**1.36[b]**	**0.4-3.16**	**0.19[a]**	**0-1.66**	**0.66[a,b]**	**0.03-1.56**	**<0.001**	**0.013**
	Lachnospiraceae		**15.13[b]**	**6.16-24.89**	**7.44[a]**	**1.17-19.26**	**13.81[a,b]**	**3.66-23.45**	**0.011**	**0.049**
		Blautia	**7.2[b]**	**2.92-17.19**	**2.88[a]**	**0.32-9.3**	**5.25[a,b]**	**1.32-10.71**	**0.035**	**0.097**
		Dorea	0	0-0.2	0.01	0-0.15	0	0-0.1	0.849	0.849
		Epulopiscium	0	0-0.07	0	0-1.03	0	0-0.27	0.765	0.785
		[Ruminococcus]	**0.68[a,b]**	**0.31-1.86**	**0.55[a]**	**0.1-3.56**	**1.57[b]**	**0.46-5.96**	**0.035**	**0.097**
		**Uncl. *Lachnospiraceae*.1**	**6.7[b]**	**2.71-14.48**	**3.08[a]**	**0.4-6.21**	**5.05[a,b]**	**1.58-8.18**	**0.002**	**0.019**
		Uncl. *Lachnospiraceae*.2	0.3	0-0.61	0.2	0.07-0.47	0.34	0.02-0.94	0.107	0.200
	Lactobacillaceae		0.69	0-67.59	0.09	0-20.13	1.01	0-47.93	0.242	0.296
		Lactobacillus	0.69	0-67.59	0.09	0-20.13	1.01	0-47.93	0.242	0.315
	Peptococcaceae		0.32	0-0.91	0.23	0-1.82	0.17	0-0.89	0.570	0.627
		Peptococcus	0.32	0-0.91	0.23	0-1.82	0.17	0-0.89	0.570	0.654
	Peptostreptococcaceae		1.17	0-11.38	0.70	0-8.22	0.23	0.03-1.46	0.094	0.189
		**Uncl. *Peptostreptococcaceae***	**1.17[b]**	**0-11.38**	**0.24[a]**	**0-8.22**	**0.16[a]**	**0.03-0.41**	**0.009**	**0.045**
	Ruminococcaceae		**0.31[a,b]**	**0-3.45**	**0.25[a]**	**0.01-2.19**	**1.99[b]**	**0-11.46**	**0.014**	**0.049**
		Faecalibacterium	**0.27[a,b]**	**0-2.41**	**0.07[a]**	**0.01-1.95**	**1.3[b]**	**0-10.15**	**0.006**	**0.038**
		Uncl. *Ruminococcaceae*	0.12	0-1.05	0.07	0-0.51	0.43	0-2.53	0.212	0.296
	Streptococcaceae		0.23	0-16.38	1.02	0-22.00	0.19	0-14.31	0.641	0.641

		Streptococcus	0.23	0-16.38	1.02	0-22	0.19	0-14.31	0.641	0.694
	Turicibacteraceae		7.86	0.14-25.78	1.48	0.11-13.28	2.56	0.33-8.39	0.167	0.263
		Turicibacter	7.86	0.14-25.78	1.48	0.11-13.28	2.56	0.33-8.39	0.167	0.272
	**Uncl. *Clostridiales*.1**		**0.17[a.b]**	**0-0.39**	**0.05[a]**	**0-0.28**	**0.19[b]**	**0.05-0.72**	**0.008**	**0.049**
		**Uncl. *Clostridiales*.3**	**0.17[a.b]**	**0-0.39**	**0.05[a]**	**0-0.28**	**0.19[b]**	**0.05-0.72**	**0.008**	**0.043**
	Uncl. *Clostridiales*.2		0.32	0-0.51	0.29	0-0.71	0.22	0-0.48	0.494	0.572
		Uncl .*Clostridiales*.4	0.32	0-0.51	0.29	0-0.71	0.22	0-0.48	0.494	0.586
	Veillonellaceae		**0[a]**	**0-0.18**	**0.01[a,b]**	**0-0.2**	**0.15[b]**	**0-1.25**	**0.009**	**0.049**
		Megamonas	**0[a]**	**0-0**	**0[a.b]**	**0-0.15**	**0.07[b]**	**0-0.12**	**0.001**	**0.013**
		Phascolarctobacterium	**0[a]**	**0-0.18**	**0[a]**	**0-0.12**	**0.08[b]**	**0-1.18**	**0.011**	**0.047**
Fusobacteriota			1.20	0.15-10.52	5.08	0.13-20.34	3.60	0.89-27.9	0.145	0.181
	Fusobacteriaceae		1.20	0.15-10.52	5.08	0.13-20.34	3.60	0.89-27.90	0.145	0.246
		Cetobacterium	0	0-0.9	0.09	0-7	0	0-0.64	0.175	0.272
		Fusobacterium	**1.2[a,b]**	**0.15-7.15**	**0.93[a]**	**0.11-13.74**	**3.47[b]**	**0.89-27.89**	**0.045**	**0.116**
		Uncl. *Fusobacteriaceae*	0	0-2.6	0.67	0-12.45	0	0-15.41	0.068	0.157
**Pseudomonadota**			**0.01[a]**	**0-0.60**	**0.27[a,b]**	**0-1.73**	**0.28[b]**	**0.04-2.14**	**0.019**	**0.087**
	Alcaligenaceae		0	0-0.20	0.14	0-1.60	0.10	0-2.10	0.051	0.124
		Sutterella	0	0-0.2	0.14	0-1.6	0.1	0-2.1	0.051	0.123
	Enterobacteriaceae		0	0-0.04	0.02	0-1.59	0.03	0-1.01	0.094	0.189
		Uncl. *Enterobacteriaceae*	0	0-0.04	0.02	0-1.59	0.03	0-1.01	0.094	0.192
	Succinivibrionaceae		**0[a,b]**	**0-0.51**	**0[a]**	**0-0.11**	**0.03[b]**	**0-0.18**	**0.013**	**0.049**
		Uncl. *Succinivibrionaceae*	0	0-0.27	0	0-0.08	0.01	0-0.16	0.107	0.200

Supplementary table 4 (S4). Fecal microbiota composition (% of relative abundance from rarefied data) at the species level including the significant results splitted by age group: Junior (J), Adult (A) and Senior (S). Different superscript letters identify significant differences among the groups (p-value < 0.05).

	Junior		Adult		Senior		J vs A vs S	
	**Median**	**Range**	**Median**	**Range**	**Median**	**Range**	**p-value**	**q-value**
f__Bifidobacteriaceae;g__Uncl. Bifidobacterium	0[b]	0-0.12	0.22[a]	0-2.09	0.06[a,b]	0-0.36	0.025	0.079
o__Clostridiales;f__;g__;s__	0.17[a,b]	0-0.39	0.05[a]	0-0.28	0.19[b]	0.05-0.72	0.008	0.036
o__Erysipelotrichales;f__Erysipelotrichaceae;g__;s__	1.36[b]	0.4-3.16	0.19[a]	0-1.66	0.66[a,b]	0.03-1.56	0.001	0.009
o__Clostridiales;f__Peptostreptococcaceae;g__;s__	1.17[b]	0-11.38	0.24[a]	0-8.22	0.16[a,b]	0.03-0.41	0.009	0.039
f__Lachnospiraceae;g__Blautia;s__	2.5[b]	0.96-6.2	0.71[a]	0.07-2.47	2.12[a,b]	0.1-3.38	0.012	0.043
f__Bacteroidaceae;g__Bacteroides;s__	0.01[a]	0-0.9	0.06[a]	0-0.25	0.47[b]	0.07-2.44	0.001	0.009
o__Clostridiales;f__Lachnospiraceae;__;__	6.7[b]	2.71-14.48	3.08[a]	0.4-6.21	5.05[a,b]	1.58-8.18	0.002	0.013
f__Fusobacteriaceae;g__Fusobacterium;s__	1.2[a,b]	0.15-7.15	0.93[a]	0.11-13.74	3.47[b]	0.89-27.89	0.045	0.123
f__Lachnospiraceae;g__[Ruminococcus];__	0.29[b]	0.12-1.2	0.02[a]	0-0.23	0.45[b]	0.2-5.63	0.000	0.005
f__Erysipelotrichaceae;g__[Eubacterium];s__biforme	1.11[b]	0.21-4.54	0.12[a]	0-1.23	0.29[a,b]	0-6.26	0.002	0.013
f__Ruminococcaceae;g__Faecalibacterium;s__prausnitzii	0.27[a,b]	0-2.41	0.07[a]	0.01-1.95	1.3[b]	0-10.15	0.006	0.030
f__Prevotellaceae;g__Prevotella;s__copri	0[a,b]	0-0.74	0[a]	0-0.19	0.03[b]	0-6.93	0.016	0.052
f__Bacteroidaceae;g__Bacteroides;__	0[a]	0-0.33	0[a]	0-0.26	0.24[b]	0.06-0.65	<0.001	0.005
f__Veillonellaceae;g__Megamonas;s__	0[a]	0-0	0[a,b]	0-0.15	0.07[b]	0-0.12	0.001	0.009
f__Bacteroidaceae;g__Bacteroides;s__plebeius	0.02[b]	0-0.19	0[a]	0-0	0.03[b]	0-0.21	0.002	0.013
f__Veillonellaceae;g__Phascolarctobacterium;s__	0[a]	0-0.18	0[a]	0-0.12	0.08[b]	0-1.18	0.011	0.042
f__Clostridiaceae;g__Clostridium;__	0[b]	0-0	0.02[a]	0-0.74	0[a,b]	0-0.29	0.028	0.082

Supplementary table 5 (S5). Correlation between SCFA (concentration and relative percentages) and the rest of the intestinal health biomarkers assessed (qPCR results, Dysbiosis Index, cCP and IgA). Significant correlation results are marked in red (p-value < 0.05) and pink (p-value < 0.01).

SCFA	*Faecalibacterium*	*Turicibacter*	*Streptococcus*	*Escherichia coli*	*Blautia*	*Fusobacterium*	*Clostridium hiranonis*	*Bifidobacterium*	*Bacteroides*	*Lactobacillus*	Dysbiosis Index	cCP	IgA
Acetate concentration	0.235	0.123	0.149	0.142	-0.081	0.106	-0.117	0.014	0.133	0.159	0.141	-0.157	-0.088
Propionate concentration	0.346	0.122	0.142	0.092	-0.029	0.041	-0.039	-0.007	0.119	0.178	0.101	-0.171	-0.083
Butyrate concentration	0.099	0.101	-0.016	0.288	-0.189	0.289	-0.071	0.284	0.213	0.097	0.019	-0.070	-0.162
Isobutyric acid concentration	0.242	0.170	0.087	0.223	-0.034	0.316	0.156	0.285	0.297	0.182	0.041	0.085	-0.374
Isovaleric acid concentration	0.184	0.095	-0.033	0.097	0.138	0.149	0.250	0.159	0.199	0.070	-0.060	0.043	-0.303
Valeric acid concentration	0.403	0.173	-0.134	0.110	0.045	0.259	0.051	0.312	0.357	0.124	-0.166	-0.169	-0.413

SCFA Total concentration	0.230	0.130	0.085	0.217	-0.131	0.207	-0.125	0.113	0.219	0.119	0.088	-0.107	-0.148
% Acetate	0.080	-0.052	0.192	-0.126	0.044	-0.222	-0.173	-0.369	-0.110	-0.020	0.175	-0.042	0.133
% Propionate	0.143	0.034	0.102	-0.128	0.078	-0.174	0.067	-0.186	-0.065	0.002	0.045	-0.087	0.094
% Butyrate	-0.150	-0.002	-0.189	0.106	-0.151	0.178	0.026	0.315	0.038	-0.005	-0.148	-0.002	-0.028
% Isobutyric acid	0.109	0.179	0.062	0.099	0.074	0.262	0.295	0.271	0.204	0.082	-0.013	0.213	-0.283
% Isovaleric acid	0.052	0.034	-0.128	-0.033	0.249	0.044	0.360	0.128	0.098	-0.009	-0.169	0.179	-0.193
% Valeric acid	0.293	0.108	-0.243	0.038	0.134	0.212	0.138	0.277	0.306	0.005	-0.270	-0.056	-0.374

Supplementary graphic (S6). Graphical bar representation of the Phylum relative abundance (% of relative abundance from rarefied data) of each age category (Junior, Adult, Senior).

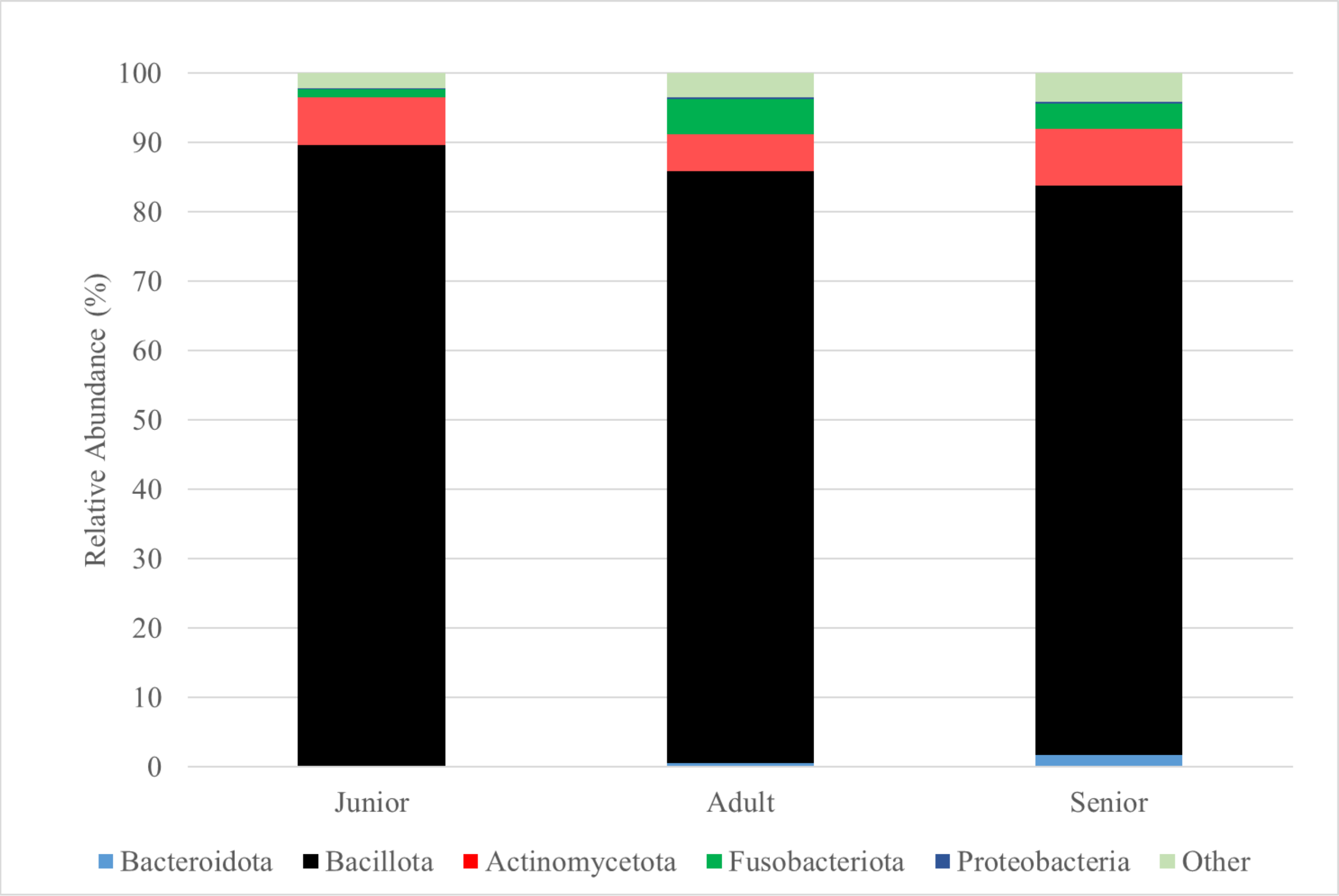

Supplement: Supplementary file 1 [file Data_Sheet_1.pdf]
